# Reconfigurable Magnetic Liquid Building Blocks for Constructing Artificial Spinal Column Tissues

**DOI:** 10.1002/advs.202300694

**Published:** 2023-07-06

**Authors:** Chao Luo, Xubo Liu, Yifan Zhang, Haoyu Dai, Hai Ci, Shan Mou, Muran Zhou, Lifeng Chen, Zhenxing Wang, Thomas P. Russell, Jiaming Sun

**Affiliations:** ^1^ Department of Plastic Surgery Union Hospital Tongji Medical College Huazhong University of Science and Technology Wuhan 430022 China; ^2^ CAS Key Laboratory of Bio‐Inspired Materials and Interfacial Science Technical Institute of Physics and Chemistry Chinese Academy of Sciences Beijing 100190 China; ^3^ Materials Sciences Division Lawrence Berkeley National Laboratory Berkeley California 94720 USA; ^4^ Polymer Science and Engineering Department University of Massachusetts Amherst Massachusetts 01003 USA; ^5^ Beijing Advanced Innovation Center for Soft Matter Science and Engineering Beijing University of Chemical Technology Beijing 100029 P. R. China

**Keywords:** structured magnetic droplets, all‐liquid molding, alginate surfactants, spinal column structures, in vivo cultivated tissues

## Abstract

All‐liquid molding can be used to transform a liquid into free‐form solid constructs, while maintaining internal fluidity. Traditional biological scaffolds, such as cured pre‐gels, are normally processed in solid state, sacrificing flowability and permeability. However, it is essential to maintain the fluidity of the scaffold to truly mimic the complexity and heterogeneity of natural human tissues. Here, this work molds an aqueous biomaterial ink into liquid building blocks with rigid shapes while preserving internal fluidity. The molded ink blocks for bone‐like vertebrae and cartilaginous‐intervertebral‐disc shapes, are magnetically manipulated to assemble into hierarchical structures as a scaffold for subsequent spinal column tissue growth. It is also possible to join separate ink blocks by interfacial coalescence, different from bridging solid blocks by interfacial fixation. Generally, aqueous biomaterial inks are molded into shapes with high fidelity by the interfacial jamming of alginate surfactants. The molded liquid blocks can be reconfigured using induced magnetic dipoles, that dictated the magnetic assembly behavior of liquid blocks. The implanted spinal column tissue exhibits a biocompatibility based on in vitro seeding and in vivo cultivating results, showing potential physiological function such as bending of the spinal column.

## Graphical abstract

Here, spinal column‐shaped blocks with biomimetic structures are fabricated by all‐liquid molding, magnetically‐driven assembly, and interfacial welding. A spinal column tissue mimic, as a proof‐of‐concept demonstration, is produced through the light‐curing of liquid blocks, in vitro seeding, and in vivo implanting into the rats.

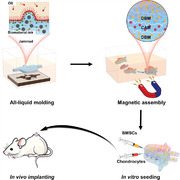

## 1 Introduction

All‐liquid molding,^[^ ^1^, ^2^ ^]^ has opened a promising path to mold aqueous solutions into permanent non‐equilibrium shapes while retaining their liquid fluidity. This technique has been developed and widely used in fabricating functional devices with a remarkable bulk fluidity and fixed surface morphology simultaneously, for example, all‐liquid circuits,^[^ ^3^ ^]^ microfluidics,^[^ ^4^ ^]^ and ferromagnetic liquid droplets,^[^ ^5^ ^]^ Here, a water droplet, containing nanoparticles, is constrained within the mold cavity, and then immersed in a more dense oil containing complementary ligands for demolding. Once floating out of the cavity, the droplet maintains the shape of the mold with high fidelity, due to a stabilization arising from the interfacial assembly and jamming of nanoparticle surfactants (NPSs) generated in situ at the water/oil interface,^[^ ^6^ ^]^ In general, the reconfigurability and stability of the liquid droplets depend on the strength of interactions between the NPs and the functionalized ligands at the oil/water interface, that is, the binding energy of the NPSs to the interface, which are responsive to external stimuli, such as pH, heat, and mechanical stirring,^[^ ^7^ ^]^ Traditional material processing in the biofabrication field makes use of curing pre‐gels or polymers,^[^ ^8^ ^]^ resulting in a loss of internal flow and permeability. Within biological scaffolds, various dispersants, for example, growth factors, nutritients, and cells,^[^ ^9^ ^]^ have to be incorporated to mimic the biological and physical microenvironment of natural tissues, which requires flow within the mimic. For instance, in 3D bio‐printing, jetted or extruded inks are immediately solidified and assembled layer‐by‐layer,^[^ ^10^ ^]^ Once solidified, it is difficult to further incorporate biomaterial inks into the printed parts. Thus, it is hard to tailor structural properties and internal flow characteristics, which ultimately limits their utility,^[^ ^11^ ^]^ Achieving tissue designs that mimic the complexity and heterogeneity of natural human tissues using all‐liquid molding is easier, since heterogeneous biomaterial inks can be injected into the liquid core within the part or mimic.

**Figure 1:**
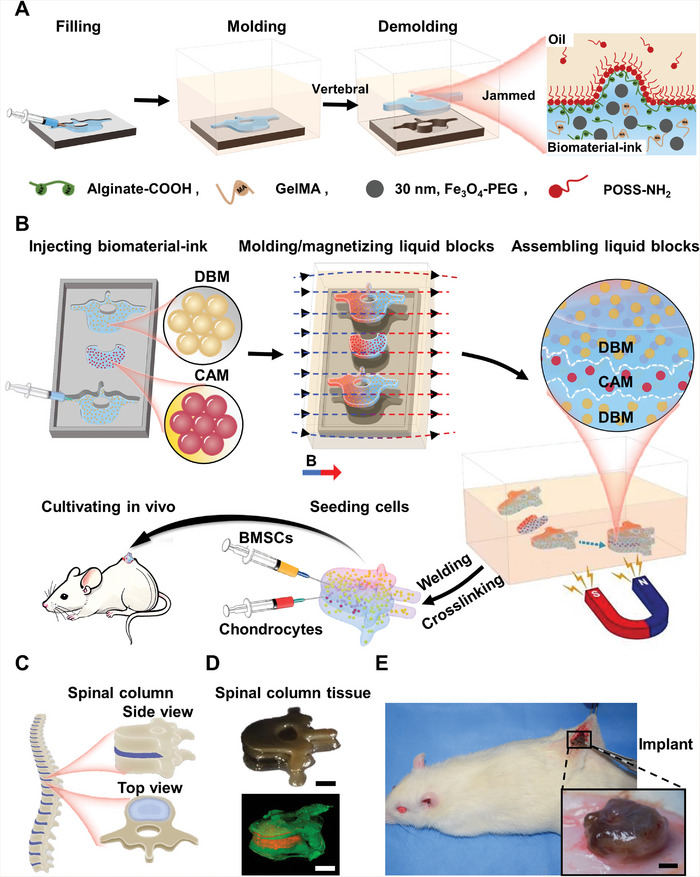
Fabricating spinal column tissue by means of all‐liquid molding techniques. A) All‐liquid molding process is illustrated. Morphologies of droplets can be retained with high fidelity after being released from the spinal column‐like mold cavity by levitating. The carboxylated alginate and amino‐functionalized POSS assemble at oil‐water interfaces until jamming, and lock the spinal column‐like shape in non‐equilibrium state. B) Molding, assembling and welding magnetic aqueous biomaterial ink into spinal column‐like droplets, seeding BMSCs/chondrocytes, blocks crosslinking, and in vivo cultivating with rats (BMSCs indicates bone marrow mensychmal stem cells, DBM indicates demineralized bone matrix, CAM indicates cartilage acellular matrix). C) Mammalian spinal columns are composed of repeating units, including vertebrae and intervertebral disc cartilage. D) Bright and fluorescent images of spinal column‐like tissue. Green color represents DBM, and red for CAM. E) In vivo cultivated spinal column tissues kept their initial shapes well after being implanted subcutaneously in the back of rats for 1 month (Scale bars in D, E: 1 mm).

Herein, we fabricated spinal column‐shaped blocks with biomimetic structures by all‐liquid molding, magnetically‐driven assembly, and interfacial welding. A spinal column tissue mimic, as a proof‐of‐concept demonstration, was produced through the light‐curing of ink blocks, in vitro seeding, and in vivo implanting into rats. Specifically, carboxylated alginate, dispersed in a water droplet, and amine‐functionalized polyhedral oligomeric silsesquioxane (POSS‐NH_2_), dissolved in the sourrounding oil, were able to assemble at the water/oil interface to generate alginate‐surfactants. Once jammed, alginate‐surfactants could form an ultrathin film with sufficient mechanical strength to stabilize the irregular shapes of the spinal column‐shaped droplets (**Figure** **1**A). Constructing the spinal column tissues includes block assembly, bridging and in vivo cultivating of vertebra‐like bone and intervertebral disc‐like cartilaginous droplet blocks (Figure 1B). In detail, 1) biomaterial ink droplets are molded into shaped blocks; 2) millimeter‐scale droplets with bone and cartilaginous shapes, floating in the surrounding oil, are manipulated magnetically to assemble into spinal column‐like structures; 3) bridging the separated blocks; 4) crosslinking by light‐curing; 5) in vitro seeding rat bone marrow mesenchymal stem cells (BMSCs) and articular chondrocytes, and in vivo cultivating in rats for 1 month.

## 2 Results and Discussion

The liquid blocks containing dispersed magnetic nanoparticles (MNPs) can be magnetized and manipulated by an external magnetic field, where particles aggregate and align into reconfigurable magnetic domains with directional moments. To cultivate spinal column tissue via magnetic biomaterial ink droplets, an acellular matrix from bone and cartilaginous tissues (demineralized bone matrix, DBM, and cartilage acellular matrix, CAM) were doped into bone‐ and cartilage‐shaped liquid blocks, respectively. The assembled spinal column‐like liquid blocks were then welded and crosslinked into hydrogel scaffolds to proliferate the cells by seeding BMSCs and chondrocytes, and cultivating for 5 days. After that, spinal column tissues were transplanted into the back of the rat subcutaneously for 4 weeks. The spinal column tissues maintained their shapes with minor deformation, and cells grew well (Figure 1C–E). This demonstrated the feasibility of fabricating the spinal column tissue, or even more complex heterogeneous tissue regeneration based on the magnetic liquid droplet blocks structured by all‐liquid molding.

We prepared composite alginate‐based “biomaterial inks,” containing polyethylene glycol (PEG)‐modified iron oxide MNPs, gelatin methacryloyl (GelMA) and specific tissue components, to realize the growth of a spinal column tissue using the molded blocks. Natural alginate and gelatin are commonly used in tissue engineering and regenerative medicine, due to their biocompatibility and biodegradability,^[^ ^12^, ^13^, ^14^ ^]^ Alginate, extracted from the cell walls of brown marine alginate, has been widely used to fabricate tissue engineered bone and cartilage grafts,^[^ ^15^, ^16^ ^]^ Interestingly, polymer chains of alginate are negatively charged, due to the large number of carboxyl groups along the backbone of the macromolecule. Therefore, alginate‐surfacants can assemble at water‐oil interfaces by the electrostatic interactions between negatively charged alginate and positively charged ligands POSS‐NH_2_ after protonation. Microgels with MNPs embedded can be manipulated remotely by an external magnetic field. Iron oxide particles are biocompatible with low toxicity,^[^ ^17^ ^]^ and photo‐crosslinking of GelMA allows the biomaterial inks to be solidified, which is essential for in vivo implanting,^[^ ^18^ ^]^

**Figure 2:**
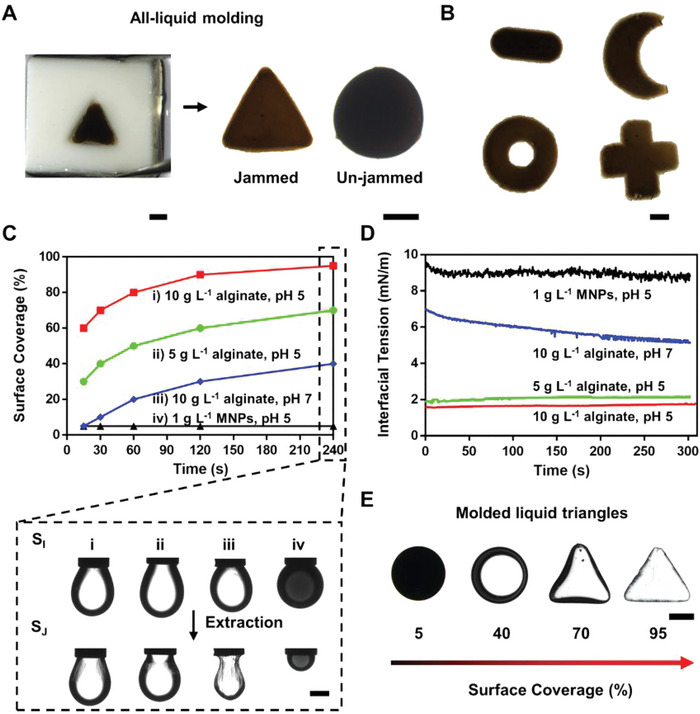
Reproducible mass production of liquid droplets with arbitrary shapes and characterization of the interfacial assembly and jamming of alginate‐surfactants. A) Triangle droplet levitated from the mold cavity immersed in the heavier oil with (Jammed) or without (Un‐jammed) POSS‐NH_2_ dissolved (Water solution: 10 g L^−1^ alginate, 100 g L^−1^ GelMA and 2.5 g L^−1^ MNPs dissolved in DI water, pH = 5; Oil: volume ratio of toluene/silicon oil/CCl_4_ is 4:3:1.). B) Liquid droplets were molded into various shapes, including cylinder, moon, doughnut and cross (Water solution: 10 g L^−1^ alginate, 100 g L^−1^GelMA and 2.5 g L^−1^ MNPs dissolved in DI water, pH = 5; 0.3 g L^−1^ POSS‐NH_2_ dissolved in oil solution where volume ratio of toluene/silicon oil/CCl_4_ is 4:3:1.). C) Surface coverage (SC) of liquid droplets were tuned by varying components, concentrations and pH, SC = S_J_/S_I_ (Oil: 0.3 g L^−1^ POSS‐NH_2_ dissolved in 100 µL toluene and 5 mL silicon oil.). D) Interfacial tension of liquid droplets were tuned by varying components, concentrations and pH (Oil: 0.3 g L^−1^ POSS‐NH_2_ dissolved in 100 µL toluene and 5 mL silicon oil.). E) The morphologies of molded liquid triangles with different SCs (Scale bars: 1 mm).

To investigate the reconfigurability and stability of the molded droplets, we characterized the interfacial assembly dynamics and apparent surface coverage (SC) of the alginate‐surfactants. The interfacial tension and SC were measured by pendant drop tensiometry. The SC was determined by S_J_/S_I_, where S_I_ is the initial surface area of the pendant droplet immersed in the oil, and S_J_ is the area when interfacial wrinkling appears when, upon decreasing the volume of the droplet, a decrease in the surface area of the droplet results, jamming the interfacial assembly. As alginate‐surfactants adsorb onto the water/oil interface, the interfacial tension decreases to ≈7 mN m^−1^ within 2 min without ligands (POSS‐NH_2_), and to ≈2 mN m^−1^ with ligands binding to the alginate‐surfactants at the interface (Figure S1, Supporting Information). The SC can be up to ≈98% within 2 min with 10 g L^−1^ of alginate at pH 5, and 3 g L^−1^ of POSS‐NH_2_ (**Figure** **2**C). The alginate‐surfactants, assembled and jammed at the oil/biomaterial ink interfaces, form a tough interfacial film that wraps the droplet and maintains the shape.

By optimizing the conditions, triangle liquid blocks could be molded as follows. A polytetrafluoroethylene (PTFE) mold containing an aqueous solution (containing 10 g L^−1^ of alginate and 2.5 g L^−1^ of MNPs) was immersed in an oil containing 0.3 g L^−1^ of POSS‐NH_2_, where the volume ratio of toluene/silicon oil/CCl_4_ is 4:3:1. The liquid droplet in the mold cavity gently rose out of the mold into the surrounding less dense oil (Figure 2A, Movie S1, Supporting Information). The extracted liquid triangle maintained its shape with high fidelity, due to the interfacial jamming of alginate‐surfacants at the droplet surface. Absent the POSS‐NH_2_ ligands, the alginate‐surfactants did not form and the liquid triangles became spherical to minimize the interfacial area (Figure 2A, Movie S2, Supporting Information).

The electrostatic attractions, between the carboxyl groups (‐COO^—^) of the alginate and terminal amino group (‐NH_3_^+^) on the ligands, were stronger with increasing deprotonation of ‐COOH and protonation of ‐NH_2_ (Figure 2C,D). The formation of alginate‐surfactant film stabilizes the shape of the liquid droplet, while the interior is still a flowable liquid, as determined rheologically. The liquid solution is still a viscoelastic fluid with a viscosity of 0.8 Pa s. (Figure S3, Supporting Information). Furthermore, we used molds of different shapes, for example, cylinders, moons, doughnuts and crosses, to shape the liquids, representing the feasibility of fabricating structured liquids with complex shape (Figure 2B). These results show that the liquids can be molded into stable shapes while maintaining their liquid character, which can be solidified for further implanting and act as a platform for cultivating spinal column tissues.

**Figure 3:**
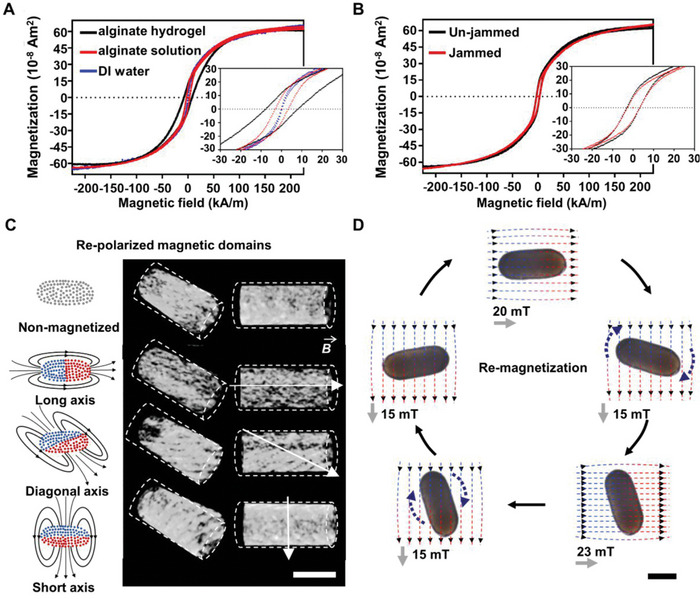
Characterization of magnetic liquid droplets. A) Hysteresis loops of individual 10 µL of liquid droplet (2.5 g L^−1^ MNPs in 10 g L^−1^ alginate solution as alginate solution, 2.5 g L^−1^ MNPs in 10 g L^−1^ crosslinked alginate solution as alginate hydrogel, 2.5 g L^−1^ MNPs in DI water as DI water) at pH 5, immersed in the oil containing 0.3 g L^−1^ of POSS‐NH_2_ ligands. B) Hysteresis loops of individual 10 µL liquid droplet (mixed with 10 g L^−1^ alginate and 2.5 g L^−1^ MNPs, pH = 5) immersed in toluene without (un‐jammed) and with POSS‐NH_2_ ligands (0.3 g L^−1^, jammed). C) Reconstructed 3D micro‐CT image of liquid cylinders with reconfigurable directional magnetic dipoles. D) A liquid cylinder was magnetized repeatedly by external uniform magnetic field. Magnetic domains and dipoles were reconfigured accordingly by applying magnetic field with specific strength and directions (Scale bars in C, D: 1 mm).

To precisely control the motion and assembly of the shaped droplets containing the iron oxide particles, an external magnetic field was used to impart a remanent magnetization to the molded liquids. Magnetic hysteresis loops of ellipsoidal liquid droplets, with different stiffnesses (2.5 g L^−1^ MNPs in DI water, 10 g L^−1^ alginate solution and 10 g L^−1^ alginate hydrogel, respectively), were measured. The remanent magnetization (*M*r) and vanishing coercive field (*H*c) are positively correlated to the internal stiffness of the droplet (**Figure** **3**A). This suggests that the dispersed MNPs are jammed by the polymer network, which transforms the paramagnetic liquid droplet into a ferromagnetic liquid droplet. We note that the MNPs are surface modified and stabilized with PEG, which can not interact with the POSS‐NH_2_ at the oil/water interface. As a result, the saturation magnetization (*M*s), *M*r and *H*c are the same regardless of interfacial absorption of POSS‐NH_2_ (Figure 3B). Therefore, MNPs only aggregate within the bulk phase and magnetize the droplets from the bulk, independent of the interface.

MNPs aggregate and arrange into magnetic domains in the direction of the applied external magnetic field. To demonstrate this, liquid cylinders were magnetized under external magnetic field in different directions (short axis, diaganol axis, long axis). These magnetized liquid cylinders were imaged by micro‐Computed Tomography (micro‐CT). 3D reconstruction of micro‐CT images showed that the distribution and orientation of high‐density area (iron oxide MNPs) were consistent with the magnetization directions (Figure 3C). Interestingly, if a stronger field was applied to the same cylinder, the magnetized cylinder would be re‐magnetized, changing the strength and direction of the original magnetic dipole moment. As illustrated, a liquid cylinder (length‐width ratio is 2.5:1) is magnetized along the long axis under a uniform 20‐mT magnetic field. Under a relatively weak magnetic field (15 mT), the magnetized liquid cylinder rotates until its long axis is parallel with the magnetic flux, followed by an orientation of the cylinder along the magnetic flux lines. If a higher magnetic field (>23 mT) is applied along the short axis, the magnetic moment of the liquid cylinder is changed and becomes parallel with the short axis of the cylinder. Under a weaker magnetic field (15 mT), the re‐magnetized liquid cylinder now rotates until its short axis is parallel to the magnetic flux lines (Figure 3D, Movie S3, Supporting Information). We also investigated the ferromagnetic behaviour of droplets with different shapes. The liquid cylinder (length‐width ratio is 5:1) and round droplets can be magnetized and re‐magnetized under the same conditions mentioned above (Figure S5, Movie S3, Supporting Information). This means liquid droplets with various shapes can be manipulated precisely with a controlled magnetic field. The magnetization and re‐magnetization of multi‐droplets were also realized by applying a magnetic field covering many droplets, thus the droplets are able to be “programmed” to move as required (Figure S6, Movie S4, Supporting Information). The translational and rotational motions of magnetized droplets are controlled by various external magnetic fields with high precision that was used to assemble the magnetic liquid blocks.

**Figure 4:**
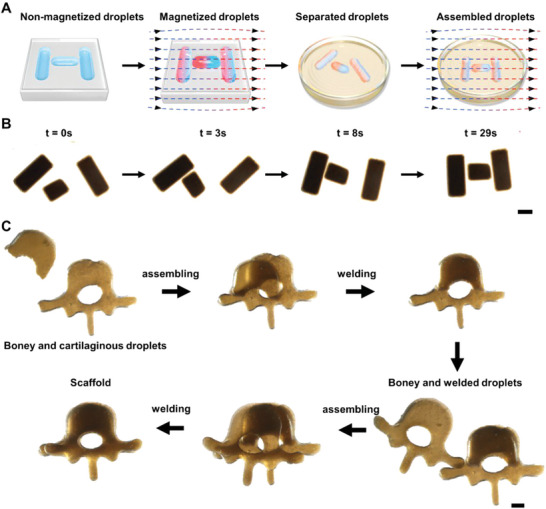
Magnetically‐controlled assembly and joint of structured liquid blocks. A,B) Formation of “H”‐shaped liquid blocks through all‐liquid molding, magnetizing and patterning. C) Bone‐like and cartilaginous liquid blocks were suspended in the oil, and manipulated by external magnetic field to assemble into a spinal column‐shaped structure. Then the gaps of droplets were welded and integrated into a scaffold for the following cell seeding and tissue growth (Scale bar in B: 2 mm, scale bar in C: 1 mm).

With a storage modulus of 4 Pa, the structured liquid blocks could keep their initial shapes (e.g., cylinders, triangles and letters) without deformation, while moving and assembling into a specific 3D pattern under an external magnetic field. As shown in **Figure** **4**A, the biomaterial ink was injected into a PTFE mold with a sculptured “H‐shaped” groove, consisting of two vertical and one transverse channels. Then, the separated cylinders of the “H‐shaped” liquids were magnetized with a 20‐mT uniform magnetic field. Once released from the mold cavity and floating in the oil, the separated cylinders, with a fixed magnetic dipole along the long and short axis, respectively, aligned along the applied field and assembled into a “H” automatically (Figure 4B, Movie S5, Supporting Information). Magnetic domains, consisting of magnetized particle strings, endowed the cylinders with a non‐contact force to move and align them. We programmed H, U, S and T as the pattern in which to assemble the cylinders, and produced the relevent continuous hydrogel structure with high fidelity (Figure S7, Movie S5, Supporting Information). For growing spinal column tissues, the separated liquid blocks were welded and gelled as a scaffold for further seeding (Figure 4C, Movie S6, Supporting Information). Various technologies have been used for the bio‐assembly of tissue blocks. For instance, acoustic‐based technology provides a contactless assembly method for tissue engineering and additive manufacturing,^[^ ^19^, ^20^, ^21^ ^]^ Traditional magnetic bio‐assembly methods constrained the assembly of the tissue blocks to be in specific patterns under the external magnetic field,^[^ ^22^, ^23^ ^]^ Here, precise assembly processes are achieved in vertical and horizontal directions by controlling the blocks magnetized with their dipoles in specific directions, which provides another tool for bottom‐up fabrication of artificial tissues.

**Figure 5:**
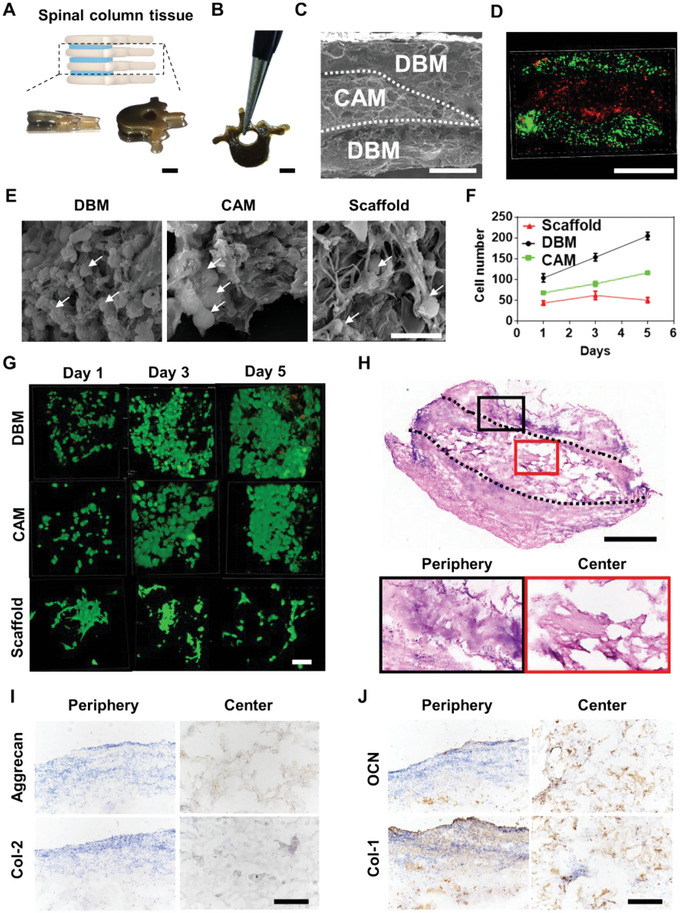
Growth and characterization of spinal column tissue. A) Bone‐like blocks containing DBM (white parts) and cartilage blocks containing CAM (blue). B) The assembled, welded and crosslinked spinal column‐like hydrogel scaffold was stable with a high mechanical strength. C) Cross‐section SEM images of spinal column‐like hydrogel scaffold indicated that the welded hydrogel blocks are closely packed with obvious boundaries between them. D) Spinal column‐like hydrogel scaffold composed of three layers of cell‐ECM synergies.(Green fluorescence: BMSCs, red fluorescence: chondrocytes). E) SEM images of seeded BMSCs/chondrocytes on spinal column‐like scaffold after culturing for 5 days. F) Quantitative analysis of the number of viable cells in different groups (White arrows indicate the seeded BMSCs/chondrocytes.). G) Calcein/PI staining of seeded BMSCs/chondrocytes on spinal column‐like scaffold, wherein green fluorescence indicated cytoplasm of live cells and red fluorescence indicated the nucleus of dead cells. H) H&E staining of explants indicated that the spinal column tissue kept its shapes. And the cells grew into the spinal column tissues in vivo with slight deformation after 4‐week implantation subcutaneously. I) Immunohistochemical staining of aggrecan and type 2 collagen indicated that the seeded chondrocytes were distributed in the center of the explant. J) Immunohistochemical staining of osteocalcin (OCN) and type 1 collagen indicated that the seeded BMSCs were distributed in the periphery area of the explant. (Scale bars in A–D and H: 1 mm; scale bars in E, G: 20 µm; scale bars in I,J: 100 µm).

For the fabrication of spinal column tissues, 1 g L^−1^ of DBM/CAM was added into the biomaterial inks for molding, that is, vertebrae‐like bone‐like and intervertebral‐disc‐like cartilaginous liquid blocks, respectively, forming a sandwiched spinal column‐like continous hydrogel structure. The top and bottom layers of the bone‐like liquid blocks and the middle layer of cartilaginous liquid blocks were assembled and solidified for in vivo implanation. The structure is biomimetic and matches natural spinal column, where the anterior is connected, half of the posterior is unattached and free to move (**Figure** **5**A). The spinal column tissue can be bent and it rebounds after compression, demonstrating potential bending and load‐bearing functions (Figure S8, Supporting Information). The three blocks are closely packed and can be easily lifted by holding the end of the upper bone‐like block with a tweezer, showing an excellent mechanical strength of the solidified scaffold (Figure 5B). Boundaries between the blocks are obvious from the the SEM images (Figure 5C). Tensile tests were performed to investigate the adhension strength of the crosslinked hydrogel assemblies. We found that stable hydrogel combinations are formed through welding and cross‐linking of the separate liquid blocks, and the concentration of alginate and fluidity of the junction points can influence the stability of the assemblies (Figure S9–S13, Movie S8, Supporting Information). To evaluate the distribution of DBM and CAM in the spinal column tissue, we stained DBM/CAM microparticles by immersing them in a solution containing 1 g L^−1^ green and red fluorescent microparticles, respectively, for 12 hrs. The green‐fluorescence labled DBM at the top/bottom and the red‐fluorescence labled CAM in the middle are easily seen in the fluorescent images (Figure 5D). This result indicates that the assembled spinal column tissue can not only simulate the native spinal column in appearance, but also achieve the biomimetic distribution of its internal tissue components while functioning, for example, bearing load and bending. Functional artificial cells or tissues are constructed by integrating biofabrication strategies with advanced material processing techniques,^[^ ^24^, ^25^, ^26^ ^]^ With the improvement of droplet manufacturing technologies, we anticipate the ability to encapsulate biological macromolecules on a smaller scale for the fabrication of artificial cells and tissues.

To investigate the potential biocompatibility of the spinal column tissue, rat BMSCs and articular chondrocytes were injected with a 32 G syringe needle in the bone‐like and cartilaginous blocks, respectively. After culturing for 5 days, SEM images and confocal images of Calcein/PI assay indicate that the number of living cells is similar to that directly seeded on DBM/CAM (Figure 5E–G). We also implanted the cultured spinal column tissue into the subcutaneous pocket in the back of a rat. After a 4‐week cultivation, the spinal column tissue maintained its shape with little deformation and was surrounded by a capsule, indicating that the “biomaterial inks” have good biocompatibility, since the surrounding in situ tissue may slowly grow onto the implanted spinal column tissue. The capsule has no obvious thickening or contraction, and it is closely connected to the surrounding tissue. H&E staining of the explant sections indicated that the cells in situ grew into the spinal column tissue during implantation. And immunohistochemical staining showed that the seeded chondrocytes were distributed in the center and the seeded BMSCs were distributed in the periphery area of the explant (Figure 5H–J). The oil‐water system is typically used in fabricating bioactive scaffolds for tissue engineering,^[^ ^27^ ^]^ The residual organic solvents can be removed from the scaffold by washing with PBS or sterilized water before cell seeding. Compared to traditional vertebral fusion or disc replacement, the spinal column tissue made by liquid blocks provides a new segmented spinal column replacement with good integration of vertebra and intervertebral disc.

## 3 Conclusions

In conclusion, we demonstrated that spinal column tissues could be constructed in magnetic liquid blocks by means of all‐liquid molding techniques. Due to the interfacial assembly and jamming of alginate‐surfacants, aqueous biomaterial inks with non‐equilibrium shapes can be molded with high fidelity and stabilized with small deformation in the oil. The magnetic dipole moment of liquid blocks can be re‐programmed and reconfigured precisely, and magnetized liquid blocks can assemble into spinal column‐like patterns as required with an external magnetic field. Liquid biomaterial inks, immobilizing tissue specific ECM, are solidified by gelation of GelMA and alginate before implanting. In vitro seeding and in vivo cultivating indicated that the seeded cells grew well on the spinal column‐like scaffolds. Tissue transplants with more biomimetic morphologies and functions, for example, artificial ear, nose and crystalline lens, are expected to be further developed by combining liquid molding techniques with existing bio‐fabrication strategies.

## 4 Experimental Section

### Materials and Reagents

Sodium alginate powders, carboxyl‐modified iron oxide mangetic nanoparticles (Fe_3_O_4_‐CO_2_H MNPs, 30 nm), Amine‐modified polyhedral oligomeric silsesquioxane (POSS‐NH_2_, Mw = 917 g mol^−1^), Silicon oil (viscosity ≈5 cSt) and 2‐hydroxy‐1‐[4‐(2‐hydroxyethoxy) phenyl]−2‐methyl‐1‐propanone (I2959) were obtained from Sigma‐Aldrich. Gelatin methacrylate (GelMA) raw material was received from EFL Biotech (Suzhou, China). Toluene (>99.8%), carbon tetrachloride (CCl_4_, >99.9%), hydrochloric acid (37%) were obtained from Sinopharm Chemical Reagent Co., Ltd (Shanghai, China). Green and red fluorescent microspheres (diameter ≈1.1 µm) were purchased from Thermo Scientific. DBM and CAM microparticles were prepared as previously reported,^[^ ^28^, ^29^ ^]^ The low/high glucose Dulbecco's Modified Eagle's Medium (DMEM), fetal bovin serum (FBS) were purchased from Hyclone. Penicillin and streptomycin were obtained from Thermo Fisher Scientific.

### Animals

Male Sprague Dawley rats (8 weeks old, 220–270 g body weight) were purchased from the Department of Experimental Animals, Tongji Medical College, Huazhong University of Science & Technology (Wuhan, China). All animal studies performed here were approved by the Department of Experimental Animals, Tongji Medical College (Wuhan, China) (2019s1154). All animal procedures were consistent with state regulations and laws and in accordance with the Standing Committee on Ethics in China (State Scientific and Technological Commission of China). All rats were sacrificed after the experiment consistent with state regulations and laws.

### All‐Liquid Molding

Polytetrafluoroethylene (PTFE) molds were designed by UG NX 10.0 software and fabricated by a digital laser puncher. Before molding, 3 µL CCl_4_ solution was injected in the mold for prewetting. Then, biomaterial ink solutions (2.5 g L^−1^ MNPs‐10 g L^−1^ alginate, pH = 5) were injected into the prewetted mold, then the mold was transfered into oil (toluene: CCl_4_: silicon oil = 4:1:3, 0.3 g L^−1^ POSS‐NH_2_). The liquid droplet was then demolded and immersed in the oil due to the high density of the oil. Images of the liquid droplets were obtained by a stereo microscope (Nikon).

### Measurement of Interfacial Tensions and Surface Coverages

Experiments of the interfacial tension between water and silicon oil (containing 100 µL toluene and 0.3 g L^−1^ POSS‐NH_2_) were estimated by a tensiometer (dataphysics, OCA 20) using the pendengt‐drop method. SC (SJ/SI) was measured using the pendant‐drop method where the SI refers to the surface area of initial droplet and SJ refers to the surface area from which the droplet began to wrinkle. All the shape changes of pendant drops were recorded as images with a 3 digital camera.

### Magnetism Characterization

Magnetic hysteresis loops of liquid droplets were measured by a vibrating sample magnetometer (Lakeshore) at 25 °C. 10 µL sphere liquid droplets containing 2.5 g L^−1^ MNPs of different stiffness were immersed in toluene with 0.3 g L^−1^ POSS‐NH_2_, and sealed in a liquid sample holder (730 935 Kel‐F liquid disposable cup, Lakeshore). For alginate hydrogel, sphere liquid droplet was placed in −20 °C for 20 min to freezing. Then, the frozen droplet was immersed in 55 g L^−1^ CaCl_2_ solution for 10 min to crosslinking. For droplets in jammed state and un‐jammed state, the droplets with volume of 10 µL were immersed in tolune with 0.3 g L^−1^ POSS‐NH_2_ and without POSS‐NH_2_.

### Rheological Characterization

Rheological properties of biomaterial ink solutions were determined using a rheometer (GÖTTFERT‐RG50) with a 40 mm cone‐and‐plate geometry, 800 µm gap at 26 °C. To explore the viscosity of biomaterial ink solutions, Shear sweeps were measured when the shear rate was changing from 0.1 to 1000 s^−1^.

### Magnetization and Re‐Magnetization of Liquid Droplets

A clamp‐like electromagnet was made to generate current‐induced magnetic fields that magnetize and guide the motion of liquid droplets. An uniform magnetic gap (55 mm × 30 mm × 20 mm) was formed between the two clamps, with a setup of the magnetic field up to 60 mT. The magnetization and re‐magnetization of liquid droplets were completed when the physical direction of the droplet was parallel to the magnetic flux, with a transient magnetic field of up to ≈20 mT and 23 mT.

### Micro‐Computed Tomography of Magnetized Droplets

A 2 µL droplet was injected into a capillary glass tube with 1 mm inner diameter and kept cylinder shape, then magnetized in different directions in the electromagnet for 5 min. The capillary tube containing the liquid cylinder was placed in the CT compartment for scanning. 3D reconstruction of the internal density distribution map was performed by CTAn software.

### Magnetic Assembly of Liquid Blocks

Biomaterial ink (1 g L^−1^ MNPs‐100 g L^−1^ GelMA‐10 g L^−1^ alginate, pH = 5) was injected into the mold and magnetized under 20 mT magnetic field in specific direction. After de‐molding, the suspended liquid blocks were controlled by an NdFeB external magnet to arrange into predetermined pattern. Then assembled liquid blocks were welded by a heated needle or increasing the ambient temperature. The welded liquid blocks were irradiated by ultraviolet (UV, 320–390 nm, 15 mW cm^−2^, ≈3 min) for crosslinking GelMA. Then, the partially crosslinked hydrogels were then removed from the oil phase and transffered into CaCl_2_ (55 g L^−1^) solution for crosslinking calcium alginate.

### Tensile Test

The adhesion properties of assembled hydogels was estimated by tensile testing. Two rectangular liquid droplets was welded and crosslinked into a dumbbell shape hydrogel of 26 mm in length, 10 mm in width and 1 mm in thickness for the tensile test. Tests were performed by using an Instron Universal Testing Machine (5967, USA) at a 10 mm min^−1^ crosshead speed and a 2 N load cell.

### Fabrication of the Spinal Column Tissue

Demineralized bone matrix (DBM) and cartilage acellular matrix (CAM) microparticles of about 100 µm were made as previously reported,^[^ ^28^, ^29^ ^]^ Then 1 g L^−1^ DBM/CAM was mixed in the aqueous solution (1 g L^−1^ MNPs‐100 g L^−1^ GelMA‐10 g L^−1^ alginate, pH = 5) as bone‐like/cartilaginous biomaterial ink. The bone‐like/cartilaginous biomaterial ink was molded in vertebra/intervertbral disc‐like liquid blocks, then assembled layber‐by‐layer under magnetic control. The liquid assemblies were then welded by heating, and irradiated by ultraviolet (UV, 320–390 nm, 15 mW cm^−2^, ≈3 min) and immersed in CaCl_2_ (55 g L^−1^) solution for 10 min to crosslinking. The crosslinnked spinal column‐like hydrogel was subjected to cryo‐gelation for 16 h in −20 °C, followed by lyophilization process for 30 min (Boyikang, Beijing, China) to fabricate porous scaffold for cell‐seeding. Rat bone marrow mesenchymal stem cells (BMSCs) and articular chondrocytes were isolated as previously reported. Before cell seeding, the lyophilized scaffolds were washed using sterilized phosphate buffer saline (PBS) three times followed by immersion in 75% ethanol for 1 h. This was followed by washing with PBS three times to remove ethanol. Then, 10^6^/mL BMSCs/chondrocytes were injected in the periphery/center of the scaffold. The cell‐seeded scaffolds were incubated at 37 °C.

### Confocal Imaging of the Spinal Column Tissue

To stain DBM and CAM microparticles, 20 g L^−1^ DBM/CAM microparticles were immersed in solutions containing 1 g L^−1^ green/red fluorescent microparticles (1.1 µm, Thermo Scientific) for 12 h. These dyed DBM/CAM microparticles were added into aqueous biomaterial ink sollutions to construct the spinal scaffold as previously described. The scaffold encapsulating dyed DBM/CAM microparticles was observed under a stereo fluorescent laser microscope (Nikon, Japan).

### Scanning Electron Microscope Imaging of the Spinal Column Tissue Construct

After proliferation for corresponding period (2 h, day 3, day 5), spinal column tissues were removed from the culture plates. After washing with PBS for three times and fixed in 4% (vol/vol) glutaraldehyde for 24 h, they were dehydrated using serial concentrations of ethanol (50%, 75%, 80%, 90%, and 100%). The dried samples were then sputtered with gold, and observed by SEM (S3400N, Hitachi, Tokyo, Japan).

### Live/Dead Assay

Calcein/ propidium iodide (PI) staining was performed to evaluate live and dead cells on scaffolds after cell seeding for 12 h, 3 days and 5 days. Briefly, samples were collected and cut through the coronal plane with a thickness of about 500 µm, washed three times with PBS. The cleaned samples were immersed in 2 µg mL^−1^ Calcein (Sigma, St. Louis, USA) and incubated at 37 °C for 30 min to stain the cytoplasm of living cells green. After washing three times with PBS, samples were immersed in 1 mL of 100 µg mL^−1^ PI (Sigma, St. Louis, USA) and incubated at room temperature to stain the nuclei of dead cells red. The stained samples were observed under a confocal laser microscope (Nikon, Japan). Cell numbers were counted over 3 pictures of randomly chosen positions from the cell‐seeded scaffolds as previously described,^[^ ^28^ ^]^

### Subcutaneous Implantation of the Spinal Column Tissue

For animal experiment, 6 male Sprague Dawley rats were used. The experimental time was about 30 min for each rat. Two samples were implanted per rat. After cell seeding for 2 h, the spinal column tissues were collected. Two subcutaneous pockets were made on the back of each rat, and spinal columns were implanted in the pockets. 4 weeks after implantation, the implanted samples were harvested and stored in −80 °C.

### H&E and Immunohistochemical Staining

The frozen samples were transferred into a Microtome‐Cryostat (Thermo Scientific, US) and cut into 10 µm sections, stained with Hematoxylin and Eosin (Sigma, St. Louis, USA) for tissue morphology examination. For immunohistochemical staining, sections were blocked by 3% BSA for 30 min, then incubated with Aggrecan, Col‐1, Col‐2, and OCN monoclonal antibodies (Abcam, Cambridge, UK). Both antibodies were diluted in PBS and then colored with diaminobenzidine tetrahydrochloride (DAB, Dako). The colored sections were rinsed with deionized water to terminate coloring. Finally, images were captured using a phase contrast microscopy (Ni‐E, Nikon, Tokyo, Japan).

## Conflict of Interest

The authors declare no conflict of interest.

## Supporting information

Supporting Information

Supplemental Movie 1

Supplemental Movie 2

Supplemental Movie 3

Supplemental Movie 4

Supplemental Movie 5

Supplemental Movie 6

Supplemental Movie 7

Supplemental Movie 8

## Data Availability

Research data are not shared.
