# Supplementary material for: Reconfigurable Magnetic Liquid Building Blocks for Constructing Artificial Spinal Column Tissues
Source: Adv Sci (Weinh). 2023 Jul 6;10(25):2300694. doi: 10.1002/advs.202300694 (PMC10477840; doi:10.1002/advs.202300694)
Supplement: Supplementary file 1 — Supporting Information [file ADVS-10-2300694-s004.pdf]

ADVANCED SCIENCE
Open Access

# Supporting Information

for *Adv. Sci.*, DOI 10.1002/advs.202300694

Reconfigurable Magnetic Liquid Building Blocks for Constructing Artificial Spinal Column Tissues

*Chao Luo, Xubo Liu, Yifan Zhang, Haoyu Dai, Hai Ci, Shan Mou, Muran Zhou, Lifeng Chen, Zhenxing Wang*, Thomas P. Russell* and Jiaming Sun**

**Title:**

**Reconfigurable Magnetic Liquid Building Blocks for Constructing Artificial Spinal Column Tissues**

**Authors**

*Chao Luo, Xubo Liu, Yifan Zhang, Haoyu Dai, Hai Ci, Shan Mou, Muran Zhou, Lifeng Chen, Zhenxing Wang*[*]*, Thomas P. Russell*[*]*, Jiaming Sun*[*]

**Dedication:** Chao Luo and Xubo Liu contributed equally to this work.

**Affiliations**

Dr. C. Luo, Dr. Y. Zhang, Dr. H. Ci, Dr. S. Mou, Dr. M. Zhou, Dr. L. Chen, Prof. Z. Wang, Prof. J. Sun

Department of Plastic Surgery, Union Hospital, Tongji Medical College, Huazhong University of Science and Technology, Wuhan 430022, China.

E-mail: sunjm1592@sina.com

Dr. X. Liu, Dr. H. Dai

CAS Key Laboratory of Bio-Inspired Materials and Interfacial Science, Technical Institute of Physics and Chemistry, Chinese Academy of Sciences, Beijing 100190, China

Dr. X. Liu, Prof. T. P. Russell

Materials Sciences Division, Lawrence Berkeley National Laboratory, Berkeley 94720, California, United States

Polymer Science and Engineering Department, University of Massachusetts, Amherst 01003, Massachusetts, United States

Beijing Advanced Innovation Center for Soft Matter Science and Engineering, Beijing University of Chemical Technology, Beijing 100029, P. R. China

E-mail: russell@mail.pse.umass.edu

**Key words**: structured magnetic droplets, all-liquid molding, alginate surfactants, spinal column structures, *in vivo* cultivated tissues

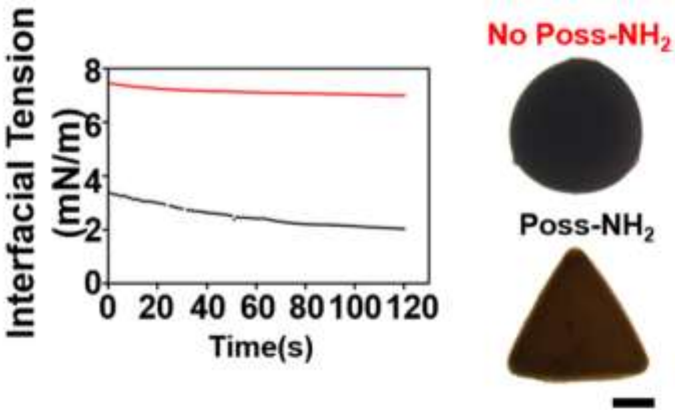


**Figure. S1.** Interfacial tension of the droplet immersed in the oil with and without 0.3 g $L^{-1}$ POSS-$NH_2$. The shape of triangle droplet buoyant in the oil can be locked by interfacial assembly and jamming of POSS-$NH_2$ and carboxylated alginate. While the droplet deformed into spherical shape without jammed layer of alginate surfactants. (Scale bar: 1 mm.)

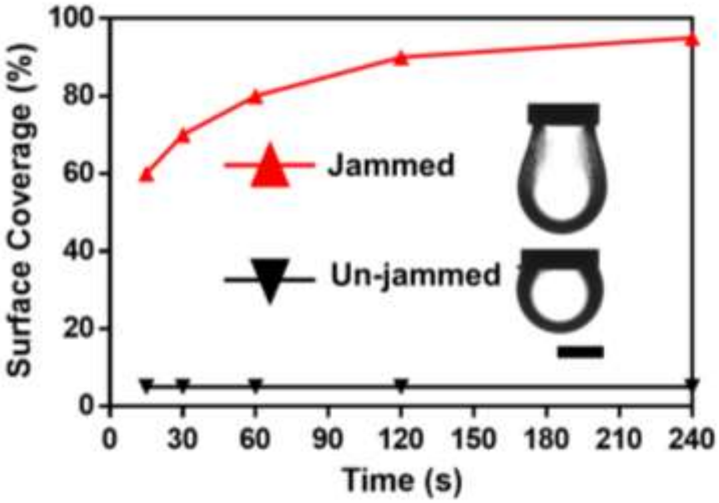


**Figure. S2.** Surface coverage of the droplet immersed in the oil with (jammed) and without 0.3 g $L^{-1}$ POSS-$NH_2$ (un-jammed). (Scale bar: 1 mm.)

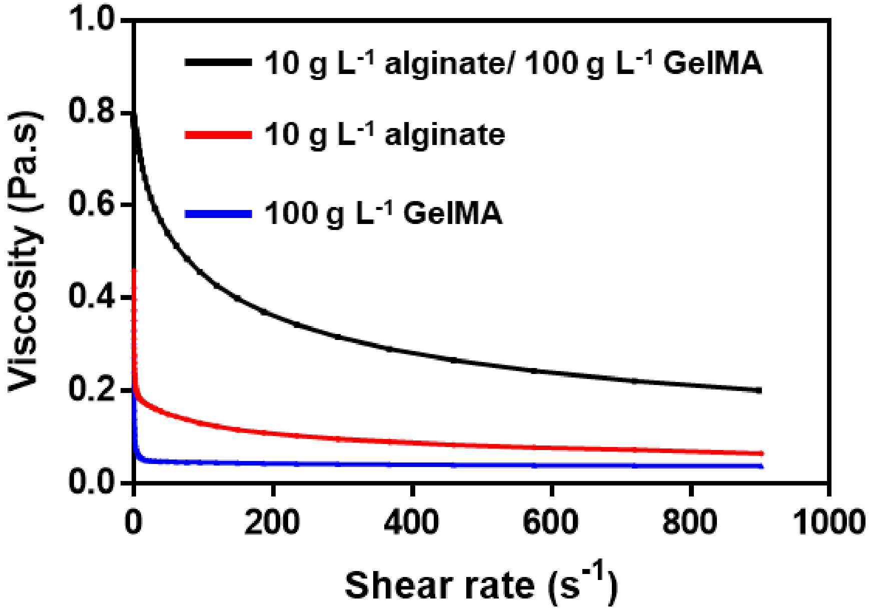


**Figure. S3.** Shear sweeps of mixture solutions containing 10 g $L^{-1}$ alginate/100 g $L^{-1}$ GelMA-10 g $L^{-1}$ alg-2.5 g $L^{-1}$MNPs/100 g $L^{-1}$ GelMA when the ambient temperature is ≈ 26 °C.

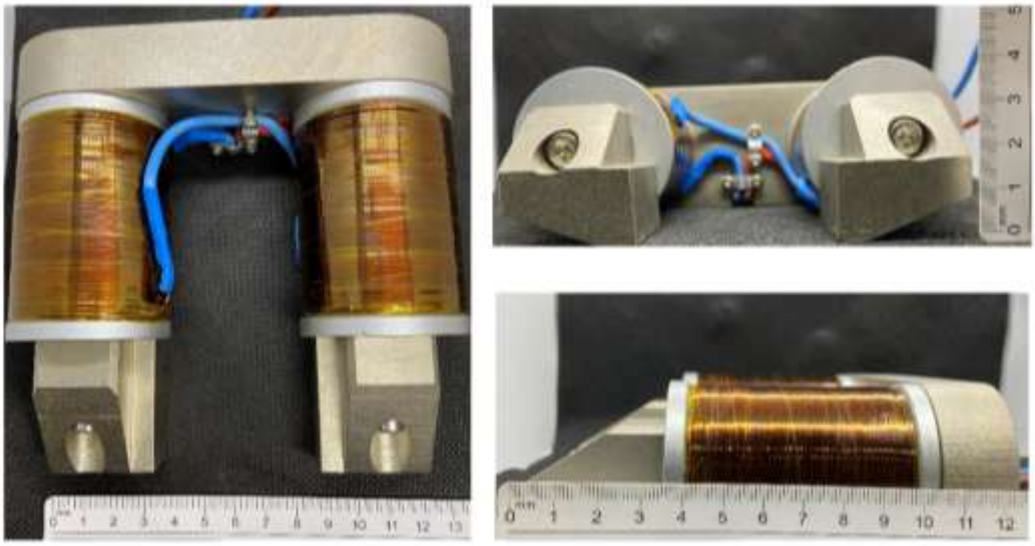

**Figure. S4.** Clamp-like electromagnet for the generation of magnetic field. Copper wire with ~0.2 mm diameter was wound into two solenoids, with the frontier extrude outside like clamps to generate current-induced magnetic fields. Once electrified, a magnetic gap forms between the two steel plates. The magnetic field in the central gap can be up to 60 mT using a current of I = 5 A.

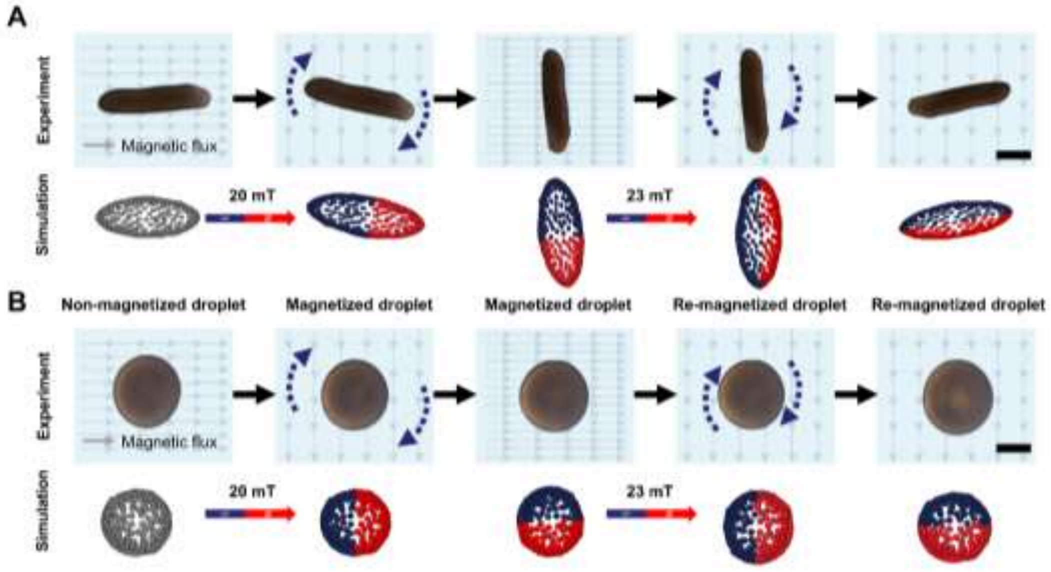


**Figure. S5.** Experiment and simulation of the magnetization and re-magnetization process of (A) liquid cylinder (Length-width ratio is 5:1) and (B) liquid sphere. The non-magnetized droplet can be magnetized under an external magnetic field up to ~ 20 mT, and remains its magnetic dipole under the control of a relatively weak magnetic field(15 mT). If a relatively strong magnetic field (up to 23 mT) is applied, the magnetic moment would be 'overwrite'. Droplets with different shapes could be magnetized and re-magnetized under same condition. (Scale bars: 1 mm.)

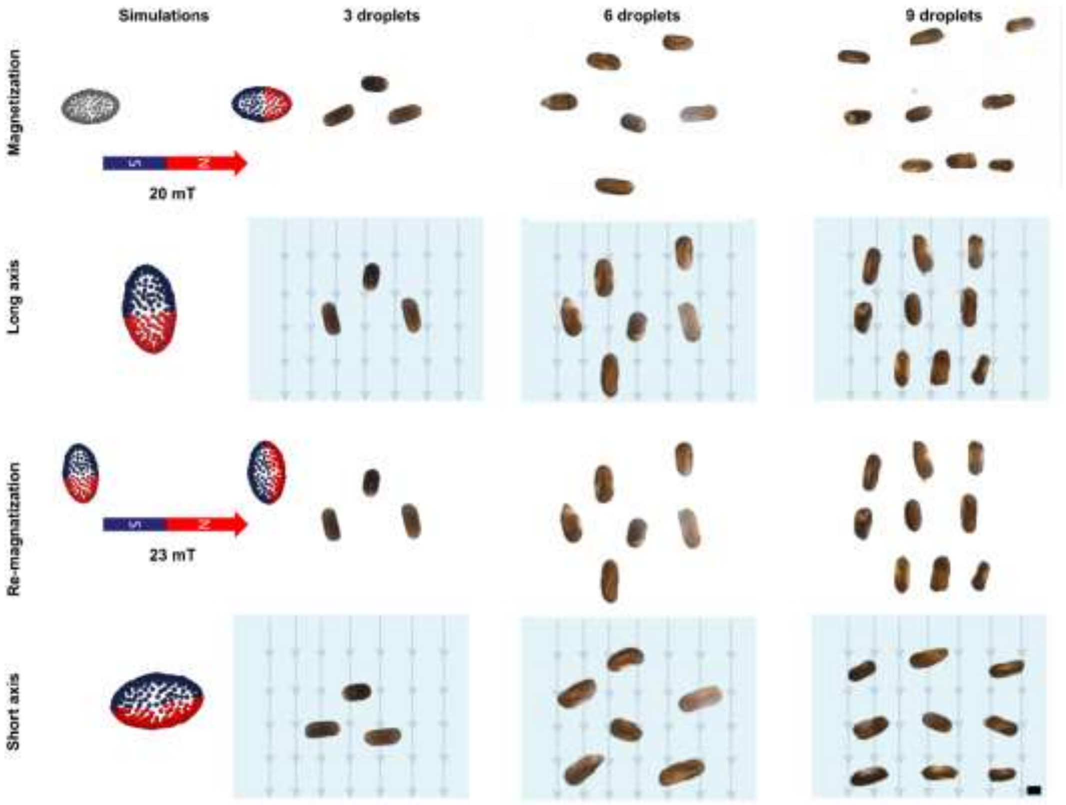


**Figure. S6.** Magnetization and re-magnetization of multi-droplets. Multi-droplets can be magnetized and re-magnetized simultaneously and move in a uniformly ordered manner under an external magnetic field. (Scale bar: 1 mm.)

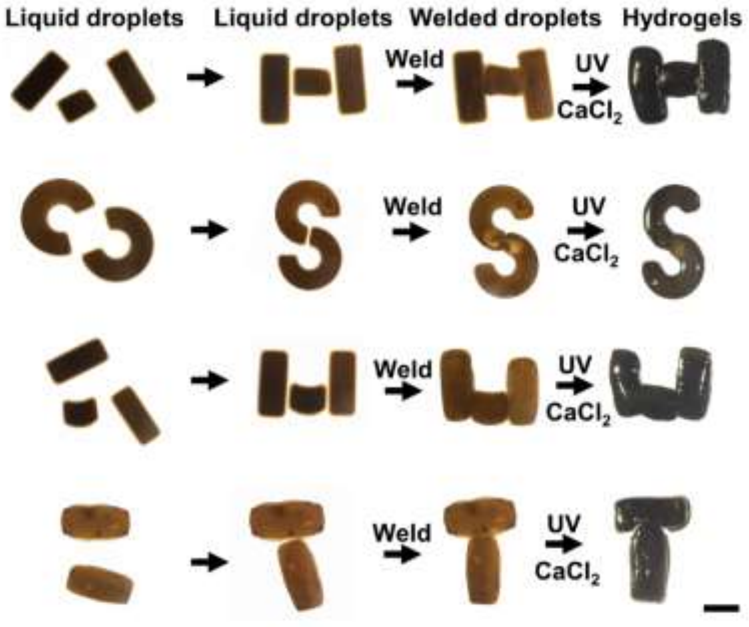


**Figure. S7.** The hydrogel letters were made by assemblying, welding and crosslinking of the liquid droplets. (Scale bar: 1 mm.)

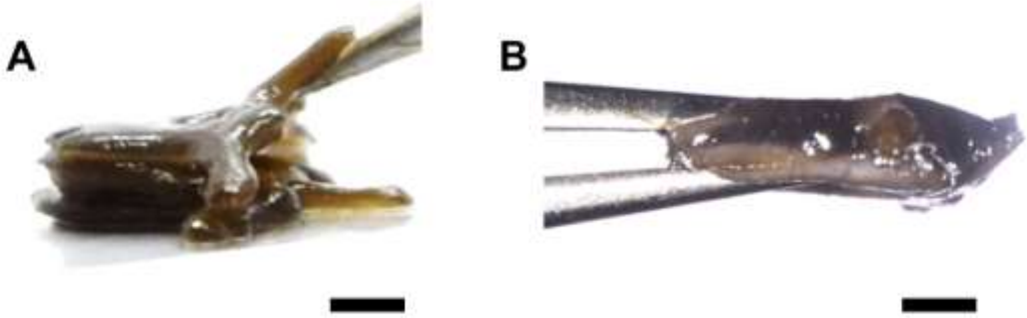


**Figure. S8.** The demonstration of potential bending and load-bearing functions of spinal column tissue. (A) The posterior of the spinal column tissue is unattached and can be bent in a maximum angle of 60 degrees. (B) The anterior of the spinal column tissue can be compressed to 2/3 of the original thickness due to the elasticity of the hydrogel. (Scale bars: 1 mm.)

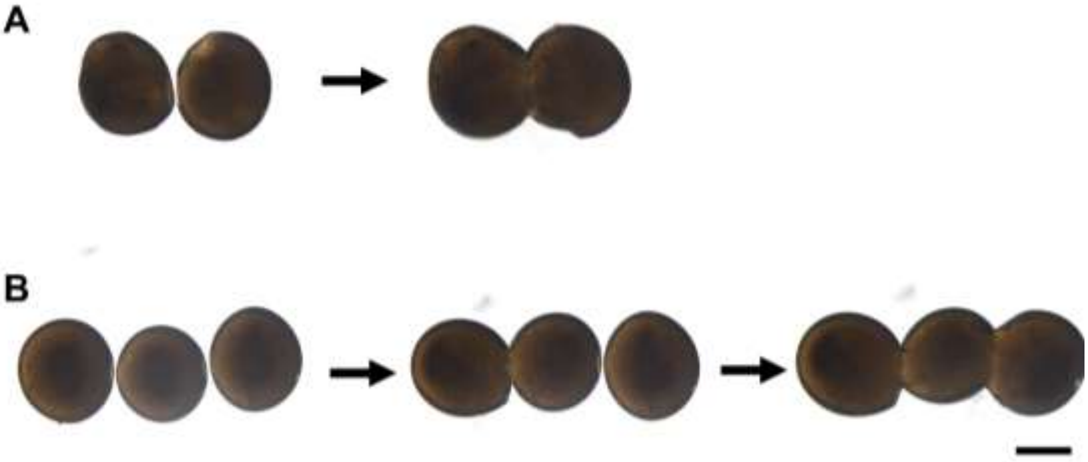


**Figure. S9.** Seperated liquid droplets were welded into continous liquid assemblies. Liquid bdidge was formed at the contacted area. The welding process was achieved by destroying the junction area through heating, thus forming a liquid bridge to merge the surrounded liquid droplets (Movie. S7). (Scale bar in A, B: 1 mm.)

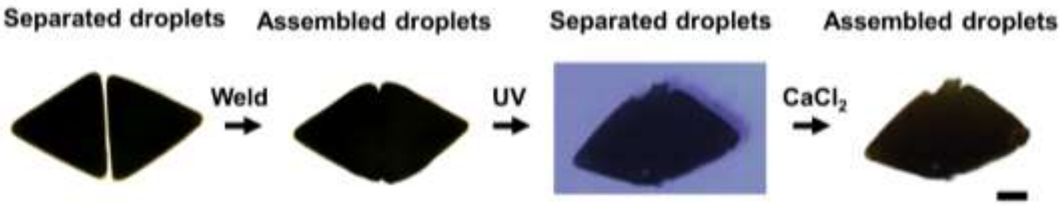


**Figure. S10.** Liquid triangles were welded into liquid rhombus, then photocroslinking GelMA irradiated under UV light and transferred into $CaCl_2$ solution for ionic crosslinking alginate. (Scale bar: 1 mm.)

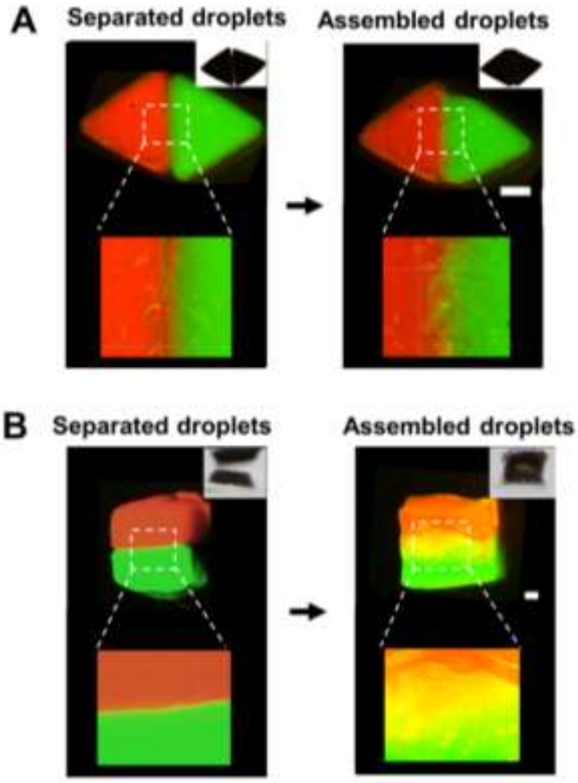


**Figure. S11.** Liquid droplets were welded in horizontal (A) and vertical (B) directions. The red and green fluorescenct dye penetrates each other through the welded boundry, indicating the formation of liquid bridge between welded liquid blocks. (Scale bars in A, B: 1 mm.)

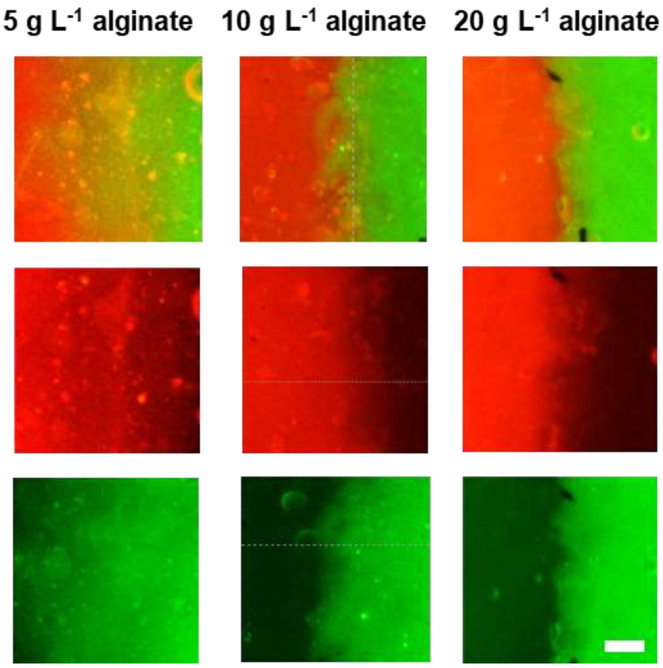


**Figure. S12.** The penetration depth decreases with higher concentration of alginate. We suppose that the alginate can increase the viscosity, so the 5 g $L^{-1}$ alg group has better liquidity and its diffusion range is deeper. (Water solution: 100 g $L^{-1}$ GelMA and 5/10/20 g $L^{-1}$ alginate dissolved in deionized water, pH = 5; Oil: volume ratio of toluene/silicon oil/$CCl_4$ is 4:3:1; scale bar: 200 μm.)

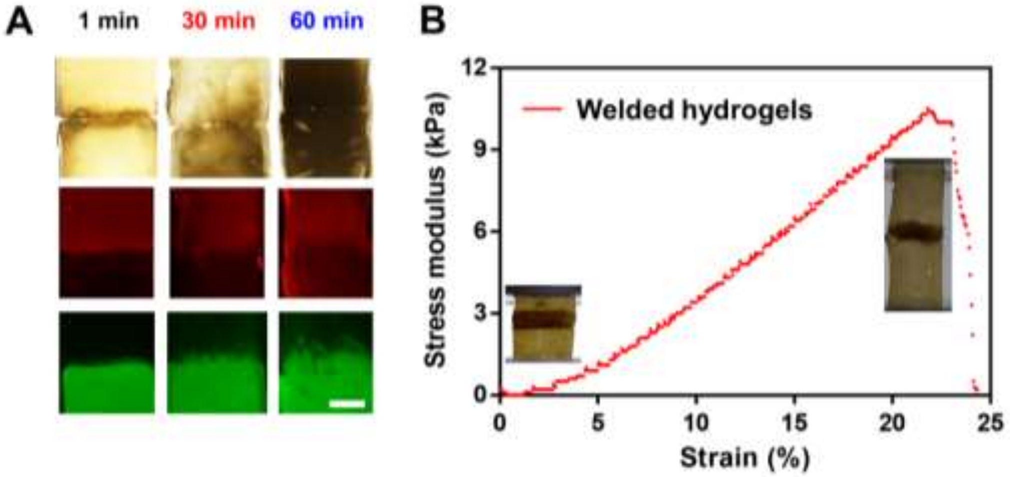


**Figure. S13.** The penetration depth increases with longer liquid diffusion time on the welded area. (A) Two liquid rectangular droplets were welded and stayed in 28 ℃ to allow liquid diffusion at the junction site. It can be seen that the diffusion distance increases gradually with time. (B) Tensil test of assemblies indicates that the hydrogel breaks when the welding area extends twice of its intial state. This result may indicate that the connection of the assembly is stable and strong. (Water solution: 100 g $L^{-1}$GelMA, 10 g $L^{-1}$ alginate and 1 g $L^{-1}$ MNPs dissolved in DI water, pH = 5; Oil: volume ratio of toluene/silicon oil/$CCl_4$ is 4:3:1.) (Scale bar in A: 1 mm.)
